# Popular Science Monthly

**Published:** 1878-10

**Authors:** 


THE POPULAR SCIENCE MONTHLY for October will be issued about September 20th. This number will open with an illustrated popular article by Prof. J. S. Newberry, of Columbia College, on "The Geological History of New York Island and Harbor," and will also contain articles by Bain, Huxley, Spencer, Kirkwood, Brooks, and other eminent home and foreign writers.

